# Temporal trend and spatial distribution of acute lymphoblastic leukemia in Iranian children during 2006-2014: a mixed ecological study

**DOI:** 10.4178/epih.e2020057

**Published:** 2020-07-29

**Authors:** Sajjad Rahimi Pordanjani, Amir Kavousi, Babak Mirbagheri, Abbas Shahsavani, Koorosh Etemad

**Affiliations:** 1 Department of Epidemiology, School of Public Health and Safety, Shahid Beheshti University of Medical Sciences, Tehran, Iran; 2 Workplace Health Promotion Research Center, School of Public Health and Safety, Shahid Beheshti University of Medical Sciences, Tehran, Iran; 3 Center for Remote Sensing and GIS Research, Faculty of Earth Sciences, Shahid Beheshti University, Tehran, Iran; 4 Environmental and Occupational Hazards Control Research Center, Shahid Beheshti University of Medical Sciences, Tehran, Iran; 5 Department of Environmental Health Engineering, School of Public Health and Safety, Shahid Beheshti University of Medical Sciences, Tehran, Iran

**Keywords:** Leukemia, Incidence, Spatial, Temporal, Iran

## Abstract

**OBJECTIVES:**

The present study investigated the spatiotemporal epidemiological status of acute lymphoblastic leukemia (ALL), the most common childhood cancer, in Iran.

**METHODS:**

Using an exploratory mixed design, this ecological study examined 3,769 under-15 children with ALL recorded in the National Cancer Registry of Iran during 2006-2014. Data were analyzed using the Mann-Whitney U test, the Getis-Ord general G (GOGG) index, optimized hot spot analysis, and Pearson correlation coefficients (PCC) at a significance level of 0.05.

**RESULTS:**

The average annual incidence of the disease was 2.25 per 100,000 under-15 children, and the cumulative incidence rate (CIR) was 21.31 per 100,000 under-15 children. Patients’ mean age was 5.90 years (standard deviation, 3.68), and the peak incidence was observed among 2-year to 5-year-olds. No significant difference was found in mean age between boys and girls (p=0.261). The incidence of ALL was more common during spring and summer than in other seasons. The GOGG index was 0.039 and significant (p<0.001). Hot spots were identified in south, central, and eastern Iran and cold spots in the north and west of Iran. The PCC between the CIR and latitude was negative (r=-0.507; p=0.003) but that between the CIR and longitude was positive (r=0.347; p=0.055).

**CONCLUSIONS:**

The incidence of ALL in Iranian children was lower than that observed in developed countries, but showed an increasing trend. It can be argued that the incidence of ALL is due to synergistic interactions between environmental, infectious, geographical, and genetic risk factors.

## INTRODUCTION

The most common type of childhood cancer, both worldwide and in Iran, is acute lymphoblastic leukemia (ALL) [1]. According to a 2014 report from the National Cancer Registry of Iran, the age-standardized incidence rate of leukemia is 6.84 per 100,000 persons [2]. With its high incidence and prevalence, leukemia leads to significant mortality and very high direct (US$15,026.6) and indirect (US$932.3) costs in Iran [3].

Leukemia is a multifactorial disease, and despite the advances in medical sciences, the main causes of its occurrence remain unknown [4,5]. The hypothesis that infectious factors play a role in the development of ALL (the infectious hypothesis) has yet to be confirmed; however, identifying seasonal patterns and spatial clusters could provide support for this possibility [6-8].

Spatiotemporal analyses are important tools for monitoring, controlling, and surveilling childhood cancer and can be very effective for generating new hypotheses [6,9,10]. Studies on spatial clustering of childhood leukemia can help identify potential sources of contamination and assist in the search for new clues about environmental risk. The question of whether leukemia shows clusters remains a scientific challenge. Previous studies have published inconsistent results; 2 studies showed no evidence of disease clustering [11,12], 2 studies observed significant clustering in at least 1 subgroup [5,13], and 6 studies found statistically significant overall clustering [4,7,9,14-16].

Controlling cancer is a top priority for health care systems. Knowledge of temporal trends, spatial patterns, and high-risk disease areas is one of the most important aspects of epidemiological studies and research into the etiology of cancer. These are essential prerequisites for health care management decision-making and the disease surveillance system.

Therefore, since the principal cause of ALL is unknown and the epidemiological status of ALL is a research gap that remains to be filled, the present study was conducted to investigate temporal trends and spatial patterns of ALL in Iran, and to acquire a better understanding of the risk factors affecting the disease cycle.

## MATERIALS AND METHODS

### Study design and type

This ecological study with an exploratory mixed design was conducted among 3,769 under-15 children with ALL in Iran from 2006 to 2014. The term “exploratory” refers to studies that explore and describe temporal trends and spatial patterns of disease incidence. This study used a mixed design, meaning that it simultaneously investigated the impact of place (a multiple-group study) and time (a time-trend study) [17].

### Target population and study population

The target population was all children with ALL in Iran. The study population included children diagnosed with ALL from 2006 to 2014 in the National Cancer Registry of Iran who met the inclusion criteria, which were a diagnosis of ALL, age less than 15 years, and accessible spatial information.

### Data collection sources

Three types of data sources were used. Patient data were obtained from the National Cancer Registry of Iran from 2006 to 2014. Data on the population of under-15 children (the population at risk) in all provinces in Iran were obtained from the Iranian Statistics Center. The geographic coordinates of the patients were obtained from the latest information available in Google Maps and georeferenced with the layer of provinces in Iran.

### Description of the study area

**Figure 1.**
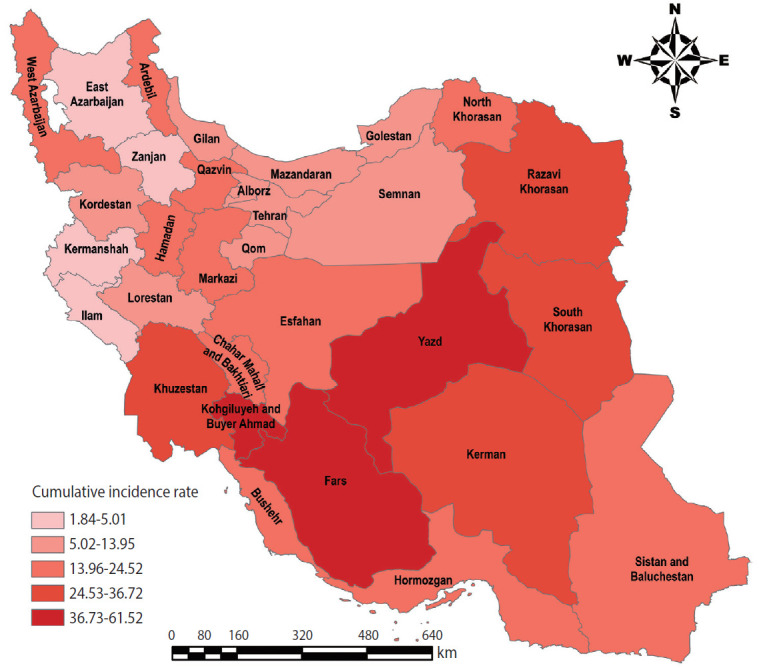
Geographical location of the study area.

Iran is a Middle Eastern country in southwest Asia with an area of 1,648,195 km^2^. Iran’s latitude extends from 25°3′ to 39°47′ north and its longitude ranges from 44°5′ and 63°18′ east (Figure 1). According to the latest census in 2016, the population of Iran is estimated to be 79,927,270.

### Statistical analysis

The statistical analysis consisted of three main parts: a descriptive analysis, a temporal trend analysis, and a spatial pattern analysis. All statistical tests were performed with a 2-sided alpha of 0.05.

### Descriptive analysis

For the descriptive analysis of data, the central tendency and dispersion indices of the disease were calculated, and then the average annual incidence and cumulative incidence rate (CIR) of ALL in each province of Iran from 2006 to 2014 were calculated.

### Temporal trend analysis

To evaluate the temporal trend of ALL, the incidence of new cases was determined by year, month, patients’ age, and patients’ sex from 2006 to 2014, and then the temporal trend was plotted and evaluated using GraphPad Prism version 8.4.2 (GraphPad, San Diego, CA, USA). The Mann-Whitney U test was used in SPSS version 16 (SPSS Inc., Chicago, IL, USA) to compare the mean age between male and female patients.

### Spatial pattern analysis and cluster identification

To evaluate the spatial patterns of ALL, the CIR from 2006 to 2014 for all provinces was first generated in ArcGIS version 10.3 (Esri, Redlands, CA, USA) from 2006 to 2014. Next, these patterns were evaluated using the Getis-Ord general G (GOGG) index and optimized hot spot analysis (OHSA).

### Getis-Ord general G

The GOGG index was developed by Getis and Ord as a global statistic for measuring spatial patterns. The GOGG index indicates the degree and amount of clustering of high and low values. If the GOGG value obtained by this method is higher than the expected value and the Z-score is positive, it indicates a tendency for high values to cluster. However, if the GOGG value is lower than expected and the Z-score is negative, it indicates a tendency for low values to cluster. The null hypothesis in GOGG is generally the absence of spatial clustering in the region [18]. In the next stage, the optimized Getis-Ord Gi* was used to identify hot spots and cold spots in different regions of Iran.

### Optimized hot spot analysis

OHSA is a more accurate, more precise, and newer method for detecting clusters. This method is based on the Getis-Ord Gi*, with the distance band optimized for hot spots and cold spots. The Getis-Ord Gi* is an index for dividing polygons based on the Z-score and p-value. If the Z-score is positive and high, it indicates the accumulation of high values and the formation of a high-risk cluster, known as a hot spot. In contrast, a negative and low Z-score indicates the accumulation of low values and the formation of a low-risk cluster, which is known as a cold spot. Next, the p-value is calculated to determine the precision and significance level of an identified cluster. For hot spots, the higher the Z-score and the lower the p-value, the less likely a cluster is to be random. For cold spots, the lower the Z-score and the lower the p-value, the less likely a cluster is to be random. If the Z-score is near zero, no spatial clustering is present. OHSA automatically selects the optimal distance band value at which clustering intensity is maximized based on the incremental spatial autocorrelation method and uses it as an analysis scale for the Getis-Ord Gi* statistic. Thus, this method shows the best possible outputs for identifying hot spots and cold spots, which can be displayed at 90%, 95%, and 99% confidence levels (Cl) [19].

### Correlation analysis

Finally, to evaluate the correlation between the CIR of ALL with the geographical coordinates of patients’ residence, Pearson correlation coefficients (PCCs) and scatter plots with a line of best fit (95% confidence interval) were used.

### Ethics statement

Ethical approval was obtained from the Shahid Beheshti University of Medical Sciences Ethics Committee (No.IR.SBMU. PHNS. REC.1398.143).

## RESULTS

### Descriptive

After applying the inclusion and exclusion criteria, 3,769 ALL patients were included. The average annual incidence of ALL was 2.25 per 100,000 children under the age of 15 years, and 57.9% (n=2,182) of the incident cases were in boys, resulting in a sex ratio of boys to girls of 1.37. The mean and standard deviation of patients’ age were 3.68±5.90 years, and the median age was 5 years. The number of incident cases and CIR of ALL in each province of Iran from 2006 to 2014 are shown in Table 1. Overall, the CIR of the disease in Iran during 2006 to 2014 was 21.315 per 100,000 children under the age of 15 years.

### Temporal trend

**Figure 2.**
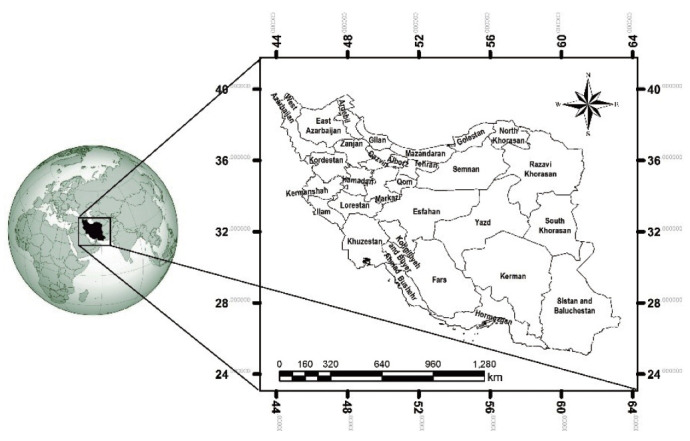
Temporal trend of incident acute lymphoblastic leukemia cases from 2006 to 2014.

The temporal trend of annual incident cases of ALL from 2006 to 2014 is shown in Figure 2. The annual number of new cases increased during this period from 269 cases in 2006 to 615 cases in 2014 (by 2.28 times). The incidence of new cases in boys was higher than in girls in all years studied.

**Figure 3.**
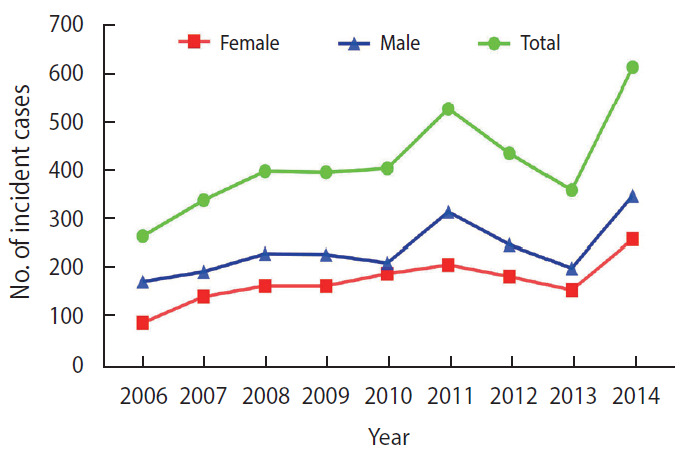
Distribution of new cases of acute lymphoblastic leukemia by age between 2006 and 2014.

Figure 3 shows the temporal trends of incident ALL cases by age. The highest incidence of new cases was in children between 2 years and 5 years of age. The graph shows that the lowest frequency of new cases was around birth (n=85). The incidence peaked at 3 years of age (n=488), and then decreased gradually from 3 years to 14 years. The age distribution of incident cases in boys and girls was right-skewed; therefore, the Mann-Whitney U test was used to compare the mean age of ALL in girls and boys. The mean age was similar in both sexes, with no statistically significant difference (p=0.261).

Seasonal variation was found in the month of diagnosis, with spring (n=1,088) accounting for more cases than summer (n=957), winter (n=896), and autumn (n=828). The highest number of new cases was reported in June (n=377), and the lowest number in November (n=242).

### Spatial pattern and cluster identification

**Figure 4.**
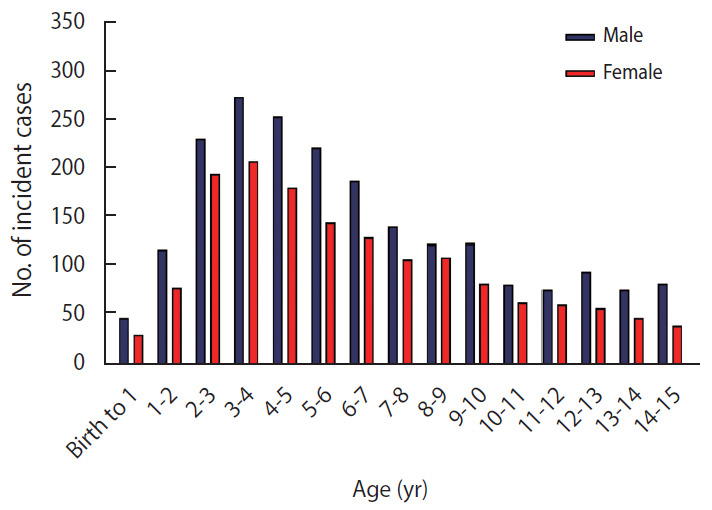
Cumulative incidence rate of acute lymphoblastic leukemia during 2006-2014 in Iran.

Figure 4 shows the spatial pattern of the CIR of ALL from 2006 to 2014.

The GOGG index was 0.039 and significant (p<0.001), indicating a tendency for high incidence rates (hot spots) to cluster.

**Figure 5.**
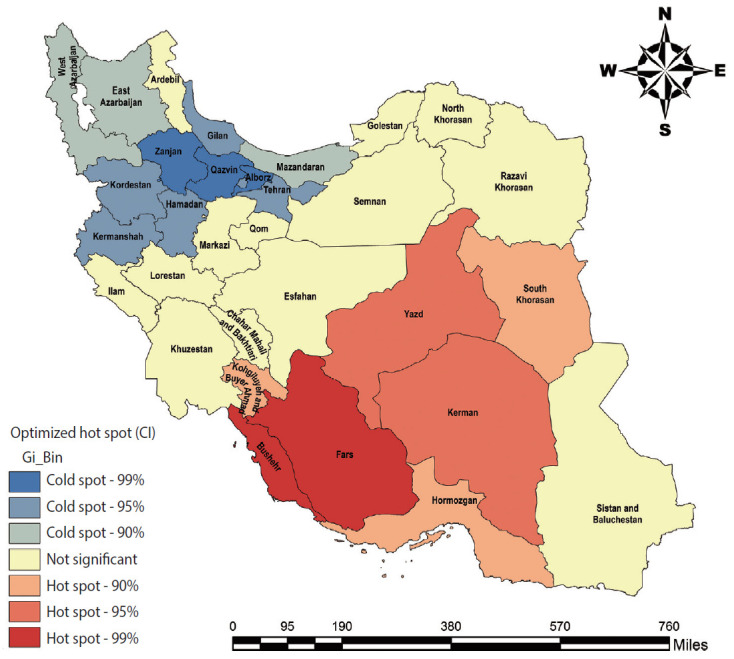
Hot spots (clusters of high values) and cold spots (clusters of low values) of acute lymphoblastic leukemia in Iran using optimized hot spot analysis. CI, confidence level.

The optimal bandwidth was estimated based on OHSA to be 448.6 km. The identified hot spots in Fars and Bushehr Provinces (99% Cl), Yazd and Kerman (95% Cl) and Kohgiluyeh and Boyer Ahmad, South Khorasan and Hormozgan (90% Cl). Moreover, it identified cold spots in Zanjan, Alborz, and Qazvin Provinces (99% Cl) and Kermanshah, Hamedan, Kurdistan, Tehran and Gilan (95% Cl) and West Azerbaijan, East Azerbaijan and Mazandaran (90% Cl) (Figure 5). All of the cold spots were located in the north and west of Iran (lower longitude and higher latitude), and all of the hot spots were located in southern, central, and eastern Iran (higher longitude and lower latitude).

The PCC between the CIR and the latitude and longitude of patients’ residence was used to assess whether ALL incidence was correlated with the geographic coordinates of patients’ residence. The PCC between the CIR and latitude was negative and significant (r=-0.509; p=0.003). Therefore, decreasing latitude was associated with a significantly higher incidence of disease. Furthermore, a positive and near-significant PCC was observed between the CIR and longitude (r=0.347; p=0.055), suggesting that increasing longitude is associated with a higher incidence of disease.

**Figure 6.**
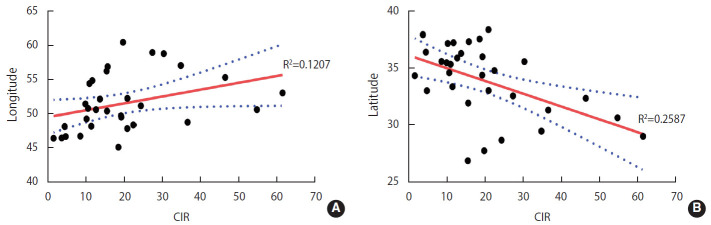
Scatter plot and line of best fit (95% confidence interval) for the correlations between the cumulative incidence rate (CIR) of acute lymphoblastic leukemia with longitude (A) and latitude (B).

A scatter plot with a line of best fit (95% confidence interval) and calculated coefficient of determination (R^2^) is shown in Figure 6.

## DISCUSSION

The average annual incidence of ALL was 2.25 per 100,000 children under the age of 15 years from 2006 to 2014. Therefore, the incidence of this disease in Iranian children was lower than has been reported in developed countries and similar to that in other developing countries. The incidence of ALL is generally higher in high-income countries with higher Human Development Index [10].

A previous study analyzed the incidence of childhood cancer in 62 countries from 2001 to 2010 and showed that the incidence of leukemia was higher in the United States, Europe, and Oceania than in Asian and African countries. It has been proposed that genetic differences may contribute to differences in the incidence of ALL, which may explain the higher incidence reported in Hispanics [20]. Overall, 4.3% of leukemia cases are attributed to an inherited genetic predisposition [21].

An analysis of the temporal trend of incident cases of ALL from 2006 to 2014 showed that the incidence of new cases has increased 2.28-fold. Similar findings have been reported in many developing countries, and may reflect the more youthful population structure of these countries [22]. It is estimated that around 70% of cases will occur in developing countries by 2030 [23].

In the present study, 57.9% (n=2,182) of cases of ALL were observed in boys, reflecting a sex ratio of males to females of 1.37. These results are consistent with other studies, which have generally shown a higher incidence of disease in boys than in girls [4,9,24-26]. According to the American Cancer Society’s 2019 report, 55% of new cases and 56% of ALL deaths were in boys [27].

In the present study, the age distribution of patients was skewed to the right. The incidence of ALL was higher in children shortly after birth (i.e., when the prevalence of pathogenic microorganisms is higher and children are more susceptible to a variety of infections due to their weaker immune system). As age increased, corresponding to strengthening of the immune system, the incidence of the disease gradually decreased. This observation implies that ALL may have an infectious origin; specifically, it may be due to an abnormal body response to common infections early in life [28-30].

The analysis of new cases based on month of diagnosis showed that ALL had seasonal variations, and that ALL was more likely to occur during the warm season (i.e., spring and summer). These seasonal variations can be considered from two perspectives: First, they are high-risk seasons for the transmission of infectious pathogens because of appropriate environmental conditions. Second, during the warm season, exposure to the sunlight is longer, meaning that exposure to solar ultraviolet (UV) radiation is increased. Both factors have been mentioned in various studies as carcinogens and risk factors for ALL. Furthermore, other studies have also reported that summer and spring were high-risk seasons for the incidence of ALL [29,31]. The seasonal pattern of ALL incidence has also been considered in some studies as a factor supporting the infectious hypothesis [8,29,32].

In a study conducted in Japan, Uehara et al. [33] reported a significant positive correlation between the risk of leukemia death and exposure to solar UVB radiation [33]. Using Poisson regression analysis, Coste et al. [34] found that for every 25 J/cm^2^ increase in solar UV radiation in children under 5 years of age, the standardized incidence rate of ALL increased significantly. Overall, there was a linear trend for solar UV radiation exposure in different geographic regions to be associated with an increase in leukemia incidence. The researchers suggested that suppression of the immune system by UV radiation may be a biological hypothesis for this finding [34].

The spatial pattern analysis showed that ALL in Iran tended to show a high spatial autocorrelation and spatial clustering, which was more intense for high rates of incidence (hot spots). The emergence of clusters in the spatial incidence of ALL has been recognized as a supporting factor for the influence of environmental risk factors, and especially infectious pathogens, on the disease cycle [7,9,13-16]. Among the predisposing, enabling, and reinforcing factors that lead to the occurrence of leukemia, some researchers suspect that viruses (e.g., hepatitis B virus, hepatitis C virus, hepatitis G virus, and human T-cell leukemia virus type 1). These viruses may contribute to the development of leukemia due to the potential for acute and chronic infections in leukocytes, especially lymphocytes (through their property of lymphotropism) [35,36].

All of the cold spots were located in the north and west of Iran (lower longitude and higher latitude) and all of the hot spots were located in southern, central, and eastern Iran (higher longitude and lower latitude). This clustering is very specific and significant, and may be due to synergistic interactions between environmental and geographic risk factors such as latitude and longitude of residence, severe exposure to solar UV radiation, and living in the oil-rich and gas-rich areas of southern Iran.

Children’s residence can serve as a reasonable proxy for environmental and local exposure, as children spend large amounts of time at home and have a low relocation rate [5]. One reason for the high incidence of ALL at low latitudes may be direct sunlight, which results in longer and more severe exposure to UV as a carcinogen that contributes to ALL. Coste et al. [34] clearly indicated that the increasing intensity of sunlight at low latitudes led to increased exposure to solar UV radiation, consequently elevating the risk of leukemia [34]. The results of the present study are in line with the results of a population-based study of childhood cancer on the Asian continent. The age-standardized incidence rate of leukemia in 0- to 14-year-old children increased with increasing longitude from West Asia to East Asia (45.2 vs. 47.4 per million person-years). The highest incidence was found in Southeast Asia, corresponding to lower latitudes (52.7 per million person-years) [20].

Another reason for the high incidence of ALL and high-risk clusters in the south of Iran could be the abundance of gas and oil resources, and the consequent high concentrations of benzene and other petroleum hydrocarbons in these areas; this possibility was recently investigated in 2 studies in 2016 and 2017 and these chemicals were reported to be a potential risk factor for leukemia [37,38].

The incidence of ALL in Iranian children is lower than that of developed countries but similar to that of developing countries. The incidence of ALL was higher in the early years after birth (peaking at age 3), when the prevalence of infections is higher and children have weaker immune systems. Given the results of the current study, including the high incidence of ALL during early years of life, seasonal variations, the tendency for high spatial autocorrelation and spatial clustering, and the significant correlation with geographical coordinates—as well as drawing upon the results of other studies—we can conclude that ALL incidence is likely to result from synergistic interactions among environmental, infectious, geographical, and genetic risk factors.

## Figures and Tables

**Table 1.** Cumulative incidence rate (CIR) of acute lymphoblastic leukemia in the provinces of Iran from 2006 to 2014

No.	Province name	No. of incident cases	Population under-15 yr	CIR per 10^6^		No.	Province name	No. of incident cases	Population under-15 yr	CIR per 10^6^
1	Alborz	56	432,664	12.943		17	Kurdistan	34	383,948	8.855
2	Ardebil	68	322,597	21.079		18	Lorestan	53	452,065	11.724
3	Bushehr	92	375,228	24.518		19	Markazi	61	313,602	19.451
4	Chahar Mahall and Bakhtiari	37	233,466	15.848		20	Mazandaran	87	623,446	13.955
5	East Azerbaijan	33	831,490	3.969		21	North Khorasan	39	243,808	15.996
6	Isfahan	213	1,008,302	21.125		22	Qazvin	45	230,359	19.535
7	Fars	620	1,007,730	61.524		23	Qom	30	276,229	10.861
8	Gilan	54	515,215	10.481		24	Razavi Khorasan	459	1,504,854	30.501
9	Golestan	53	442,656	11.973		25	Semnan	15	133,436	11.241
10	Hamadan	93	410,381	22.662		26	Sistan and Baluchestan	176	880,028	19.999
11	Hormozgan	108	686,490	15.732		27	South Khorasan	45	163,526	27.519
12	Ilam	7	139,818	5.007		28	Tehran	249	2,443,481	10.190
13	Kerman	239	684,095	34.937		29	West Azerbaijan	143	761,508	18.779
14	Kermanshah	8	435,241	1.838		30	Yazd	113	242,802	46.540
15	Khuzestan	433	1,179,140	36.722		31	Zanjan	12	252,213	4.758
16	Kohgiluyeh and Buyer Ahmad	94	171,480	54.817		Total population of Iran		3,769	17,681,629	21.315
